# The epidemiology of invasive pneumococcal disease in the Canadian North from 1999 to 2010

**DOI:** 10.3402/ijch.v72i0.21606

**Published:** 2013-08-05

**Authors:** Melissa Helferty, Jenny L. Rotondo, Irene Martin, Shalini Desai

**Affiliations:** 1 Centre for Immunization and Respiratory Infectious Diseases, Public Health Agency of Canada, Ottawa, ON, Canada; 2 Bacteriology and Enteric Diseases Program, National Microbiology Laboratory, Public Health Agency of Canada, Ottawa, ON, Canada

**Keywords:** *Streptococcus pneumoniae*, Canadian circumpolar region, surveillance, epidemiology, vaccine preventable disease

## Abstract

**Introduction:**

The International Circumpolar Surveillance network is a population-based surveillance system that collects data on invasive pneumococcal disease (IPD) in Northern Canada. A 7-valent pneumococcal conjugate vaccine was first introduced in some regions of Northern Canada in 2002, followed by 10-valent (2009) and 13-valent (PCV-13) vaccines (2010). A 23-valent polysaccharide (PPV-23) vaccine was first introduced in 1988 for special populations and adults aged 65 years and older. To describe the epidemiology in the context of pneumococcal vaccination programs, we analysed surveillance data from Northern Canada from 1999 to 2010.

**Methods:**

A standardized case report form capturing demographic and clinical information was completed for all IPD cases in Northern Canada meeting the national case definition. Isolates were sent to a reference laboratory for confirmation, serotyping and antimicrobial resistance testing. Both laboratory and epidemiological data were sent to the Public Health Agency of Canada for analysis. Population denominators were obtained from Statistics Canada.

**Results:**

From 1999 to 2010, 433 IPD cases were reported (average 36 cases per year). Incidence was greatest among infants aged <2 years and among those aged 65 years and older, with an average annual incidence of 133 and 67 cases per 100,000 population, respectively. After a peak in incidence in 2008, rates among infants have declined. Incidence rates varied from 2 to 16 times greater, depending on the year, among Aboriginals compared to non-Aboriginals. Hospitalization was reported in 89% of all cases and the case fatality ratio was 6.0%. Clinical manifestations varied, with some patients reporting >1 manifestation. Pneumonia was the most common (70%), followed by bacteremia/septicaemia (30%) and meningitis (8%). Approximately, 42% of cases aged <2 years in 2009 and 2010 had serotypes covered by the PCV-13. In addition, the majority (89%) of serotypes isolated in cases aged 65 years and older were included in the PPV-23 vaccine.

**Conclusion:**

IPD continues to be a major cause of disease in Northern Canadian populations, with particularly high rates among infants and Aboriginals. Continued surveillance is needed to determine the impact of conjugate pneumococcal vaccine programs. Additional studies investigating factors that predispose infants and Aboriginal peoples would also be beneficial.

The International Circumpolar Surveillance (ICS) network was created in 1999 to establish a surveillance network throughout Arctic countries that would provide a means of assessing, monitoring and analysing population-based rates of infectious diseases for public health action (1). In 2000, it was adopted as an Arctic Council Sustainable Development Working Group Project, which promotes coordination and cooperation among Arctic nations. Canada is an active member in this surveillance network and has 6 participating regions, including the Northwest Territories, Yukon, Nunavut, the Québec Cree and Nunavik regions and Northern Labrador. The ICS region is distinct from the rest of Canada in its demography, with 57% of the population self-declared as Aboriginal (First Nation, Métis or Inuit) compared to 4% in Canada overall. In 2011, the ICS population was estimated to be about 149,475 people, approximately 0.4% of the Canadian population. Over the years, the scope of diseases investigated by this system has expanded; beginning with surveillance of invasive disease due to *Streptococcus pneumoniae* in 1999 to now include other bacterial infectious agents such as *Neisseria meningitidis* and *Helicobacter pylori*.

Invasive pneumococcal disease (IPD) is caused by the Gram-positive bacteria *S. pneumoniae*. Invasive disease may present with a variety of manifestations, which are dependent on the site of infection, and may include meningitis, bacteremia and pneumonia (2). Previous research studies have indicated that incidence rates are elevated among Aboriginal children as compared to non-Aboriginal children (3, 4). Ninety-two serotypes have been identified globally, of which 15 cause the majority of diseases.

In Canada, 4 pneumococcal vaccines have been licensed and introduced. Between July 2002 and January 2007, the 7-valent pneumococcal conjugate vaccine (PCV-7) was introduced into childhood immunization schedules in Northern Canadian regions. This was followed by the 10-valent pneumococcal conjugate vaccine (PCV-10), which was introduced between June 2009 and February 2010. Finally, the 13-valent pneumococcal conjugate vaccine (PCV-13) was introduced between July 2010 and January 2011. All 3 vaccines were introduced as provinces and territories adopted them into their routine immunization programs. Serotypes covered in PCV-7 include 4, 6B, 9V, 14, 18C, 19F and 23F. PCV-10 covers those in PCV-7 as well as an additional 3 serotypes (1, 5 and 7F). PCV-13 covers all serotypes in PCV-10 and also includes serotypes 3, 6A and 19A.The schedule recommended by the National Advisory Committee for Immunization for all 3 conjugate vaccines has been consistent, with a primary series of 3 doses given at 2, 4 and 6 months and a booster at 12–18 months. The 23-valent pneumococcal polysaccharide vaccine (PPV-23) is used for targeted populations such as seniors and high-risk groups (5). Serotypes covered by PPV-23 include 1, 2, 3, 4, 5, 6B, 7F, 8, 9N, 9V, 10A, 11A, 12F, 14, 15B, 17F, 18C, 19A, 19F, 20, 22F, 23F and 33F.

Given that *S. pneumoniae* is a leading cause of pneumonia and meningitis in the circumpolar north among Aboriginal persons, and that changes in the epidemiology have been observed in other Arctic countries pre- and post-PCV-7 implementation (6), continued surveillance of this invasive disease is important. Therefore, the objective of this study was to describe the epidemiology of IPD in the context of pneumococcal vaccination programs in Northern Canada from 1999 to 2010.

## Methods

Using the ICS network of public health personnel and laboratories, all cases of IPD with *S. pneumoniae* isolated from a normally sterile site (excluding the middle ear and pleural cavity) who resided in any of the 6 participating regions from 1999 to 2010 were identified and included in this study. For a detailed description of the ICS network, see Ref. 1.

Isolates from confirmed cases were sent to one of 3 references laboratories: the National Centre for Streptococcus, the National Microbiology Laboratory (NML), or the Laboratoire de santé publique du Quebec (LSPQ) for serotyping and antimicrobial susceptibility testing. Serotyping was performed using the Quellung reaction using pool, group, type and factor commercial antisera from SSI Diagnostica; Statens Serum Institute, Copenhagen, Denmark. All 3 laboratories participate in on-going quality control testing. Antimicrobial susceptibilities to cefotaxime, ceftriaxone, chloramphenicol, clindamycin, erythromycin, levofloxacin, penicillin, tetracycline, trimethoprim/sulfamethoxazole, vancomycin were determined by microbroth dilution (Sensititire STP6F, Thermo Fisher Scientific, Ottawa, ON) in accordance with the Clinical and Laboratory Standards Institute (CLSI) guidelines (7, 8).

Epidemiological information was collected by a communicable disease officer in participating regions using a standardized case report form, which includes data such as demographic information, self-identified Aboriginal status, risk factors for disease (e.g. asthma, smoking), relevant immunization information, outcome and clinical manifestations. Both the laboratory and epidemiological data was forwarded to the Center for Immunization and Respiratory Infectious Diseases (CIRID) at the Public Health Agency of Canada (PHAC) for data entry, cleaning, collation and analysis. Laboratory and epidemiological data were entered into an Access database and verified by each participating region during annual data audits.

Population estimates were obtained using Statistics Canada's annual census subdivision and territorial July 1st estimates. These estimates were obtained on an annual basis. Aboriginal population estimates were developed for each year using data from the 2006 Canadian Census. Excel 2010 and SAS version 9.1 were used for the analysis.

## Results

**Fig. 1:**
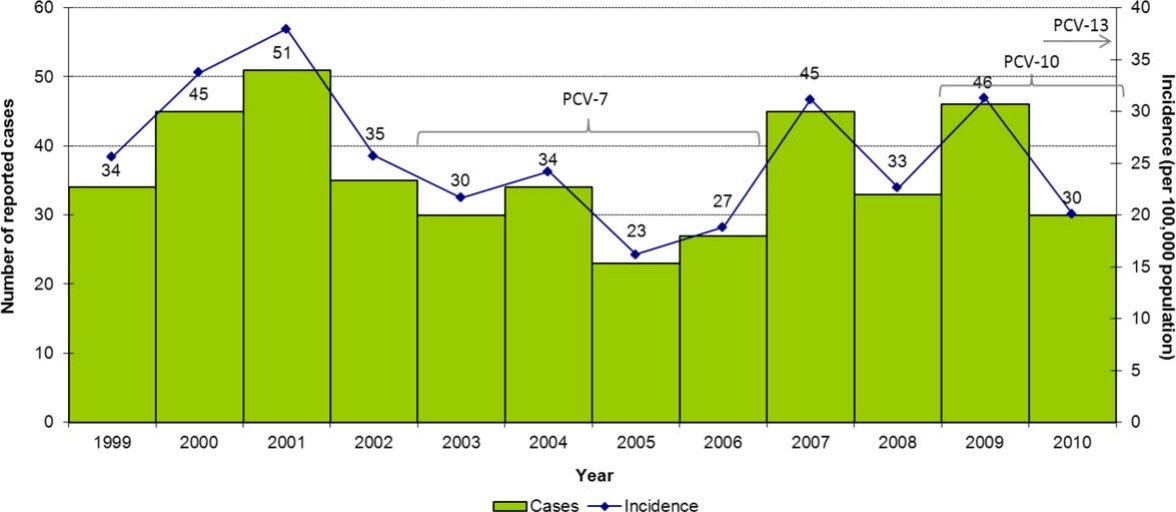
Reported cases and incidence rate (per 100,000 population) of invasive pneumococcal disease in Northern Canada by year, 1999–2010.

From 1999 to 2010, a total of 433 IPD cases were reported through the ICS system in Northern Canada (average of 36 cases per year). Figure 1 depicts the number of reported cases and incidence rates throughout this surveillance period. The average annual incidence was 25.8 cases per 100,000 population; however, the number of cases and incidence rates fluctuated, from a low of 23 (16.2 per 100,000 population) in 2005 to a high of 51 (37.9 per 100,000) in 2001.

**Fig. 2:**
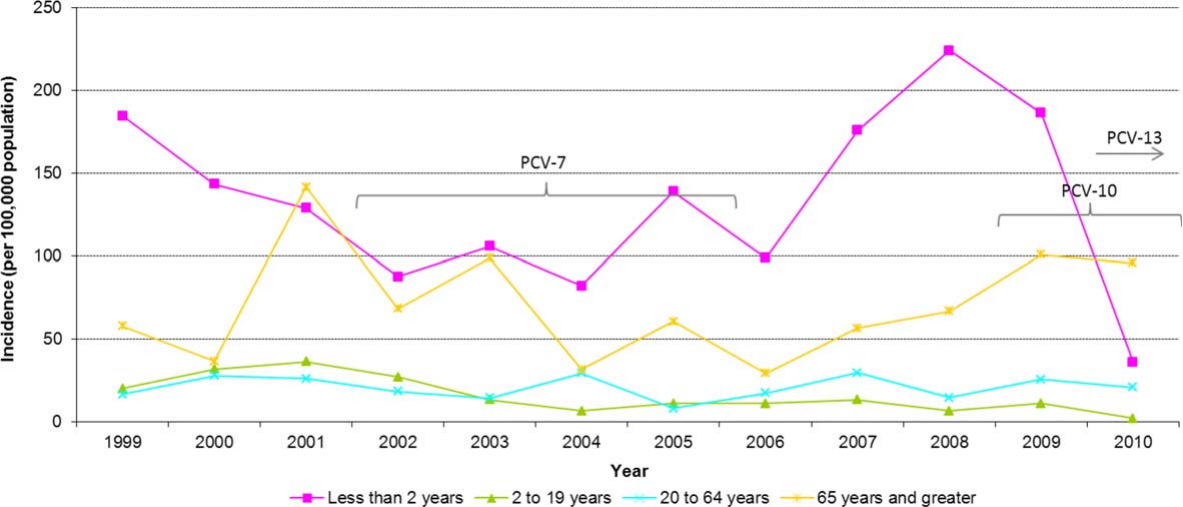
Incidence rate (per 100,000 population) of invasive pneumococcal disease in Northern Canada by age group and year, 1999–2010.

As presented in Fig. 2, incidence varied by age group with the highest average annual incidence rate occurring in infants aged <2 years (132.8 cases per 100,000 population). Incidence rates among infants aged <2 years showed a steady decline from 1999 to 2004 when they reached a low of 82.3 cases per 100,000 population. After this, incidence began to climb to a high of 224.1 cases per 100,000 population in 2008. Since then, rates have sharply declined to 26.5 cases per 100,000 population in 2010. On average, infants aged <2 years made up 19% of all IPD cases from 1999 to 2010.

The age group with the second greatest burden of illness was those aged 65 years and older, with an average annual incidence rate of 66.9 cases per 100,000 population. The incident rate decreased from 2001 to 2006 but has been on the rise since then. On average, adults aged 65 years and older comprised 13% of IPD cases each year. Cases in the 2–19 and 20–64 age groups had an average annual incidence of 15.4 and 20.2 cases per 100,000 population, respectively. Rates for these two age groups have been relatively stable over the years.

From 1999 to 2010, 75% of cases occurred in self-identified Aboriginals, 14% occurred in non-Aboriginals and 11% had an unknown ethnicity. Table 1 presents the incident rates by ethnicity for each year. The average annual incidence rate among Aboriginals was 33.7 cases per 100,000 population and was higher compared to the average incidence rate of 8.5 cases per 100,000 among non-Aboriginals throughout the surveillance period. Rates among Aboriginal cases fluctuated from a low of 18.3 cases per 100,000 population in 2005 to a high of 57.6 cases per 100,000 population in 2001. In each year, rates among Aboriginals were greater than non-Aboriginals and depending on the year, ranged from 2 to 16 times higher.

**Table 1:** Incidence rate (per 100,000 population) of invasive pneumococcal disease in Northern Canada by ethnicity and year, 1999–2010

Year	Aboriginal	Non-Aboriginal	Unknown	Total
1999	41.1	3.6	0.0	25.6
2000	41.1	18.0	2.2	33.7
2001	57.5	3.6	3.0	37.9
2002	31.6	10.5	2.9	25.7
2003	23.7	8.6	4.3	21.7
2004	29.5	10.1	2.8	24.2
2005	18.3	8.3	2.1	16.2
2006	23.0	8.2	2.1	18.8
2007	38.5	13.0	3.5	31.1
2008	28.7	6.4	3.4	22.6
2009	40.3	8.0	4.8	31.3
2010	31.7	3.1	0.7	20.1

Clinical manifestation of cases varied with some reporting more than one manifestation. Pneumonia was reported most frequently (70%), followed by bacteremia/septicaemia (30%), meningitis (8%), empyema (3%) and septic arthritis and cellulitis reported in 1% of cases.

Severity of illness was assessed using hospitalization and mortality information. A total of 366 cases were hospitalized throughout the surveillance period, representing 89% of cases with hospitalization data. In addition, 26 deaths were reported, resulting in an average annual case fatality ratio of 6%. The ratio fluctuated from no deaths reported in 2000 and 2003 to a high of 15.2% in 2009. The highest overall case fatality ratio of 20% was reported in cases aged 60 years and older, followed by 6% in cases aged 20–64 years of age. The overall case fatality ratio among cases aged <2 years of was 4% and no deaths were reported among cases between 2 and 19 years of age. There was no association observed between serotype and outcome.

Antibiotic susceptibility testing was carried out on 94–100% of isolates, depending on the antibiotic tested. The majority of isolates were susceptible to penicillin, ceftriaxone, vancomycin, clindaymycin, erythromycin, trimethoprim/sulfamethoxazole and choloramphenicol; however, some isolates did show intermediate or full resistance to trimethoprim/sulfamethoxazole (9%), erythromycin (6%), penicillin (5%), ceftriaxone (2%) and clindamycin (2%).

**Fig. 3:**
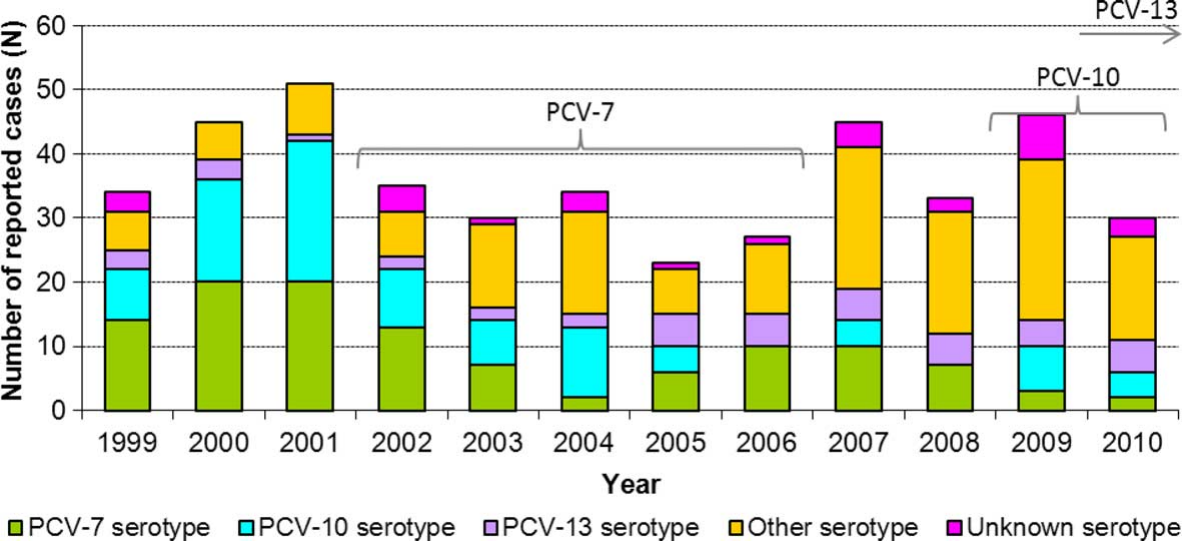
Reported cases of invasive pneumococcal disease by serotype category* in Northern Canada by year, 1999–2010. *PCV-7 serotype encompasses cases with serotypes 4, 6B, 9V, 14, 18C, 19F, and 23F. PCV-10 serotype encompasses cases with serotypes included in PCV-10 but not PCV-7 (i.e. serotypes 1, 5, and 7F). PCV-13 serotype encompasses cases with serotypes included in PCV-13 but not PCV-10 (i.e. serotypes 3, 6A, and 19A). Other serotype encompasses cases with serotypes not included in any conjugate vaccine formulation.

The distribution of serotypes by conjugate vaccine category from 1999 to 2010 is presented in Fig. 3. The proportion of cases that occurred as a result of a serotype included in PCV-7 (4, 6B, 9V, 14, 18C, 19F, 23F) decreased throughout the early 2000s to a low of 6% in 2004, after which it increased to 37% of cases in 2006. Since then, it has decreased, with only 7% of cases due to PCV-7 in 2010. The proportion of cases with one of the 3 serotypes covered by PCV-10 but not PCV-13 (1, 5, 7F) has decreased during the study period, from a high of 43% in 2001 to 0% in 2006. These serotypes accounted for 13% of cases in 2010. The proportion of cases with a serotype covered by PCV-13 but not PCV-10 (3, 6A, 19A) was greatest in 2005 (22%) but has decreased slightly (17%) since 2010.

Overall, 37% of cases had a serotype covered by one of the 13 serotypes contained in PCV-13 in 2010. Similarly, on average 42% of cases among infants aged <2 years had a serotype covered by PCV-13 from 2009 to 2010. The proportion of cases in all age groups with serotypes not included in the vaccine ranged from 13 to 20% from 1999 to 2002; since 2008, >50% of cases had serotypes not included in the vaccine. The most common serotypes from 2007 to 2010 were 8, 7F and 19A, making up 14, 8 and 7% of cases with known serotypes, respectively.

Among those aged 65 years of age and older, from 1999 to 2006 an average of 93% of cases had serotypes that were included in PCV-13; however, in 2008 and 2009 only 23% had a PCV-13 serotype. From 1999 to 2010, an average of 89% of cases aged 65 years and older had a serotype covered by PPV-23 (i.e. serotypes 1, 2, 3, 4, 5, 6B, 7F, 8, 9N, 9V, 10A, 11A, 12F, 14, 15B, 17F, 18C, 19A, 19F, 20, 22F, 23F and 33F) each year. Of the cases with a serotype included in PPV-23, 63% had received at least one dose of the vaccine 1–21 years prior to symptom onset, 25% had not been previously vaccinated with PPV-23, and 12% had an unknown vaccination history.

## Discussion

Within Canada, there are a limited number of surveillance systems that capture both laboratory and epidemiological information on vaccine preventable diseases. The ICS system continues to be an excellent example of this partnership and is fundamental to the understanding of the changing epidemiology of IPD in the Arctic. The ICS system has demonstrated that IPD continues to be an important cause of morbidity in Northern Canada. Incident rates in the North were 1.5–4.5 times greater than national rates, depending on the year, throughout this surveillance period (9). Infants, adults aged 65 years and older, and Aboriginals are particularly affected.

Within the study period, the Aboriginal population was disproportionately affected each year, with rates 2–16 times greater than those of non-Aboriginal persons. These findings correspond with other research studies that have identified a greater burden of IPD in Aboriginal populations (6, 10, 11). Several studies have cited multiple possible risk factors such as crowded living conditions, socio-economic status, underlying medical conditions and a greater exposure to risks such as smoking (6, 10, 11) as possible explanations.

Throughout this surveillance period, the greatest burden of illness was observed in young infants followed by the elderly. Although these age groups also had the greatest burden of illness in Canadian data, rates within the ICS population surpassed national rates. In children aged <1 year, the difference in the ICS region was 4–13 times greater in the period 2005–2010 (9).

Vaccine coverage assessments in the Canadian North continue to be a challenge, resulting in difficulties determining the potential role of inadequate coverage (12). Current national survey methodologies have several limitations, including the use of random digit dialling (thus excluding households without a telephone), relying on parental recall (therefore potentially underestimating coverage) and a sample size within the Canadian North that is too small to allow for provincial coverage estimates (12). The lack of information on coverage results in difficulties assessing the role of under-immunization in IPD incidence in the Canadian North and warrants further research.

Since 1999, the number of IPD cases caused by a serotype covered by a pneumococcal conjugate vaccine has decreased. At the same time, there has been an increase in cases with serotypes not covered by a conjugate vaccine. At this time, it is unclear whether this change is due to serotype replacement versus natural fluctuations in circulating strains. For cases aged 65 years and older, the majority of disease was due to serotypes covered by the PPV-23 vaccine. It should be noted that PCV-13 has been licensed for use in adults in Canada, and the National Advisory Committee on Immunization is currently reviewing the evidence for its use in this population. Further research on the uptake of pneumococcal vaccines and waning immunity in adults aged 65 years and older should be conducted.

### Limitations

The Canadian ICS region represents only 0.4% of the Canadian population and is located in the Arctic North; therefore, the generalizability of this analysis may not be applicable to other parts of Canada and other industrialized countries. The small number of cases and small population sizes need to be taken into consideration when interpreting results, as even one case can cause great variation from year-to-year incidence rates. Data on risk factors may not have been adequately captured, thereby limiting our understanding of predisposing factors for infection. In addition, not all cases had complete hospitalization and death data, which may underestimate the severity of illness observed during this study period. It is unknown whether antibiotics were administered prior to specimen collection, which may result in an underestimate of the true number of cases. Finally, information captured on vaccination history was insufficient to determine if vaccination failures occurred.

## Conclusions

IPD is an important infection that causes significant morbidity and mortality in the Canadian North, especially in high-risk populations, such as infants, the elderly and Aboriginal persons. Further analysis regarding the impact of conjugate pneumococcal vaccines in reducing IPD incidence in the Canadian ICS region is needed. On-going surveillance will provide necessary information regarding IPD epidemiology, circulating serotypes, the severity of illness, and antibiotic resistance. Further studies investigating factors that predispose infants and Aboriginal peoples would also be beneficial. On-going use of the PPV-23 vaccine among the elderly is recommended as are immunization coverage surveys in the North.
